# Association between Self-Reported Habitual Snoring and Diabetes Mellitus: A Systemic Review and Meta-Analysis

**DOI:** 10.1155/2016/1958981

**Published:** 2016-01-19

**Authors:** Xiaolu Xiong, Anyuan Zhong, Huajun Xu, Chun Wang

**Affiliations:** ^1^Department of Endocrinology, Drum Tower Clinical Medical College of Nanjing Medical University, 53 North Zhongshan Road, Nanjing 210008, China; ^2^Department of Respiratory Diseases, The Second Affiliated Hospital of Soochow University, 1055 Sanxiang Road, Suzhou 215004, China; ^3^Department of Otolaryngology, Shanghai Jiao Tong University Affiliated Sixth People's Hospital, Otolaryngology Institute of Shanghai Jiao Tong University, 600 Yishan Road, Shanghai 200233, China; ^4^Department of Geriatrics, Drum Tower Clinical Medical College of Nanjing Medical University, 53 North Zhongshan Road, Nanjing 210008, China

## Abstract

*Aim*. Several studies have reported an association between self-reported habitual snoring and diabetes mellitus (DM); however, the results are inconsistent.* Methods*. Electronic databases including PubMed and EMBASE were searched. Odds ratios (ORs) and 95% confidence intervals (CIs) were used to assess the strength of the association between snoring and DM using a random-effects model. Heterogeneity, subgroup, and sensitivity analyses were also evaluated. Begg's, Egger's tests and funnel plots were used to evaluate publication bias.* Results*. A total of eight studies (six cross sectional and two prospective cohort studies) pooling 101,246 participants were included. Of the six cross sectional studies, the summary OR and 95% CI of DM in individuals that snore compared with nonsnorers were 1.37 (95% CI: 1.20–1.57, *p* < 0.001). There was no heterogeneity across the included studies (*I* ^2^ = 2.9%, *p* = 0.408). When stratified by gender, the pooled OR (95% CI) was 1.59 (1.20–2.11) in females (*n* = 12298), and 0.89 (0.65–1.22) in males (*n* = 4276). Of the two prospective studies, the pooled RR was 1.65 (95% CI, 1.30–2.08).* Conclusions*. Self-reported habitual snoring is statistically associated with DM in females, but not in males. This meta-analysis indicates a need to paying attention to the effect of snoring on the occurrence of DM in females.

## 1. Introduction

Diabetes mellitus (DM) is becoming a major global public health problem. Compared with 1980s, the proportion of diabetes of both men (9.8% versus 8.3%) and women (9.2% versus 7.5%) increased in 2008 [1]. It is worthwhile to note that over 438 million individuals are expected to be at risk of developing DM by 2030 [2]. Besides its high prevalence, growing research also suggests that DM could induce adverse health implication [3, 4]. Similarly, habitual snoring is a common and early symptom of obstructive sleep apnea (OSA) and affects ~33% of the general population [5]. Snoring has long been considered a nuisance especially for bed partners and brought social burden (i.e., traffic accident and poor school performance) [6–8]. An increasing amount of evidence has also suggested that habitual snoring might be associated with health-related complications, including endothelial dysfunction, vascular injury, stroke, and cardiovascular diseases [6, 9–12].

Many studies have assessed the association between self-reported habitual snoring and DM. However, conflicting results have been reported. Importantly, habitual snoring might help doctors identify individuals at a higher risk of developing DM. Therefore, the relationship between these two common diseases should be evaluated comprehensively.

To our knowledge, a quantitative analysis evaluating the association between self-reported habitual snoring and DM susceptibility is not available. Thus, it is essential to perform a meta-analysis to clarify this potential association. The purpose of the current study was to identify the association between self-reported snoring and DM by performing pooled risk estimates.

## 2. Materials and Methods

We performed this meta-analysis according to the recommendations of Meta-analysis Of Observational Studies in Epidemiology (MOOSE) statement [13].

### 2.1. Literature Search Strategy

To identify studies that assessed the relationship between self-reported snoring and susceptibility to DM, we searched electronic databases systemically, including PubMed and EMBASE in May 2015. No language and human study restriction were imposed. The following combinations of MeSH terms and text word terms were used (snoring or snorer or self-reported snoring) and (diabetes or diabetic or diabetes mellitus or hyperglycemia). We also checked the reference lists of relevant articles that might be appropriate for inclusion in the meta-analysis. The searches were conducted by Drs. Xiong and Zhong, respectively.

### 2.2. Inclusion and Exclusion Criteria

Studies that met the following criteria were included: (1) studies that evaluated self-reported snoring and the risk of DM; (2) prospective observational, retrospective, cohort, cross-sectional, or case-controlled studies; (3) subjects without a diagnosis of DM at baseline or who were excluded from the final statistical analysis; (4) odds ratios (ORs) or risk ratios (RRs) and 95% confidence intervals (CIs) which were provided for comparing snorers to nonsnorers; (5) snoring status which was defined using the question “To the best of your knowledge, do you snore now or have you snored previously?” and habitual snoring which was defined by each study; (6) the definition of diabetes which was mainly according to a history of diagnosis made by a physician, fasting serum glucose levels, oral glucose tolerance test (OGTT), or the use of medicine to treat DM. Studies were excluded if (1) it was not a full-text paper (i.e., no reviews, abstracts, letters, or comments); (2) snoring was not measured using a questionnaire; (3) it analyzed subjects with gestational diabetes mellitus; (4) no variables were adjusted.

### 2.3. Data Extraction

Two authors (Drs. Xiong and Zhong) extracted the data from the included studies to a standard sheet independently. The data extracted includes the first author and year of publication, country of origin, source of the study, study design, sample size, percentage of female participants, age range, OR or RR with 95% CI, adjusted variables, the measurement used to assess snoring, and the diagnosis of DM. If there were discrepancies in the basic information of the included studies, a third reviewer (Dr. Xu) examined the inconsistent extracted data. If there were additional queries or if further details were needed, the authors of the original studies were contacted via email.

### 2.4. Quality Assessment

The methodological quality of all included studies was assessed according to the Newcastle-Ottawa scale (NOS) guidelines (http://www.ohri.ca/programs/clinical_epidemiology/oxford.asp). A study was awarded a maximum of one star for each numbered item within the selection and outcome categories. Therefore, a maximum of four, three, and two stars were given for selection, outcome, and comparability, respectively. More stars indicated a higher quality study. We recognized one star as one score, and the score of each study was presented in Table 1.

### 2.5. Statistical Analysis

ORs, RRs, and 95% CIs were used to assess the relationship between self-reported snoring and DM across the included studies. The ORs were transformed into RRs using the formula RR = OR/[(1 − *P*_*o*_)+(*P*_*o*_ *∗*OR)] (*P*_*o*_ is the incidence of the outcome of interest in the nonexposed group). If an included study reported various adjustments for covariates, the most fully adjusted OR/RR was used in pooled analysis. Heterogeneity was examined using Cochrane *Q* tests and the *I* ^2^ statistic [14]. If between-study heterogeneity existed (*p* < 0.10 or *I* ^2^ > 50%), a random-effects model was used; otherwise a fixed-effect model was applied [15]. Subgroup analysis was performed to assess the effect of significant group differences according to gender. Sensitivity analysis was performed to test the robustness of the results by omitting one study each time. Potential publication bias was assessed using Begg's rank correlation and Egger linear regression tests [16]. Unless stated otherwise, *p* < 0.05 was considered to be statistically significant. All the above-mentioned statistical analyses were performed using the STATA software (version 12.0, Stata Corp., College Station, TX, USA).

## 3. Results

### 3.1. Literature Search Results

**Figure 1:**
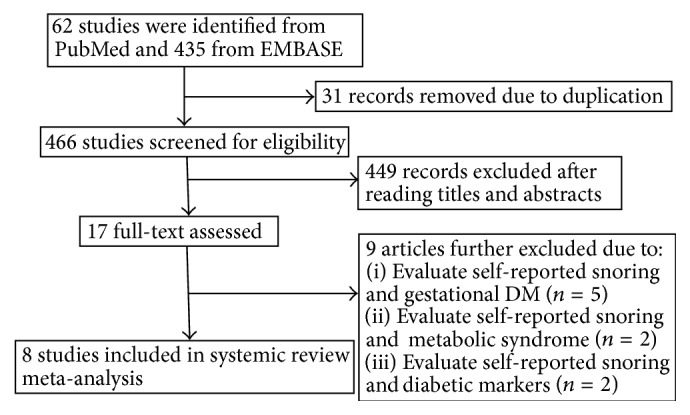
Flow chart of the literature search and study selection process.

A total of 497 potentially relevant references were retrieved from the PubMed and EMBASE databases. 31 duplicates were removed. After first reviewing the titles and abstracts, 449 studies were further excluded for various reasons. The remaining 17 papers were reviewed fully, and a further nine reported papers were excluded for evaluating self-reported snoring and gestational DM (*n* = 5), self-reported snoring and metabolic syndrome (*n* = 2), and self-reported snoring and hemoglobin A1c levels, glucose, and insulin metabolism (*n* = 2). Finally, eight studies were enrolled in the qualitative synthesis, among which six were included in the meta-analysis. The detailed search process is presented in Figure 1.

### 3.2. Characteristics of the Included Studies

A total of eight studies (including six cross-sectional and two prospective cohort studies) pooling 101,246 participants were included. The mean age of the participants at baseline ranged from 20 to 85 years. The percentage of female subjects ranged from 48.8% to 100%, except for one study that enrolled only male subjects. Three of the included studies were performed in the United States [17, 22, 23], three were conducted in Sweden [1, 18, 21], and one was undertaken in each of Finland [19] and Italy [20]. All the included studies were observational and population-based; six were designed as cross-sectional studies, and the other two [1, 23] were prospective cohorts. The follow-up duration of the two prospective studies was 10 years. Three of the included studies got a high score of eight, and the other five studies scored seven in quality assessment. The detailed properties of the studies are summarized in Table 1.

### 3.3. Pooled and Subgroup Analysis: Snoring and Risk for DM

**Figure 2:**
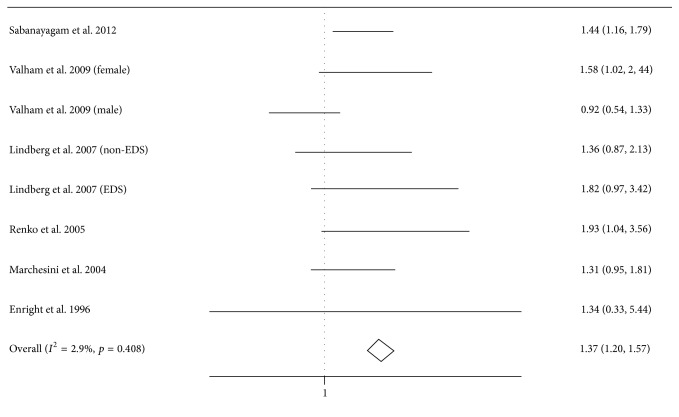
Forest plot of the association between self-reported habitual snoring and diabetes mellitus risk. Valham et al.'s study provided the data in female and male population not the whole population, and Lindberg et al.'s study provided the data in excessive daytime sleepiness (EDS) and non-EDS snorers.

In this meta-analysis, we summarized the mostly adjusted ORs and 95% CIs by pooling the six cross-sectional studies containing 28,890 subjects. Of the six studies, one study provided the data in female and male population not the whole population [18], and the other study provided the data in excessive daytime sleepiness (EDS) and non-EDS snorers [21]. The OR and 95% CI of DM in individuals with self-reported habitual snoring compared with nonsnorers were 1.37 (95% CI, 1.20–1.57, *p* < 0.001) (Figure 2). There was no heterogeneity across the included studies (*I* ^2^ = 2.9%, *p* = 0.408).

In subgroup analysis of the six cross-sectional studies, only two studies stratified according to gender [18, 20], and one study was conducted in a female population [21]. In the female population (*n* = 12298), the pooled OR was 1.59 (95% CI, 1.20–2.11), whereas OR = 0.89 (95% CI, 0.65–1.22) in the male population (*n* = 4276) [18, 20].

For the two prospective cohort studies, one study [23] was conducted in a female population of 69,852 subjects; the RR was 1.63 (95% CI, 1.29–2.07). The second study [1] was conducted in a male population of 2,504 subjects, and the RR was 1.06 (95% CI, 0.36–3.1) and 7.0 (95% CI, 2.9–16.9) in nonobese and obese populations, respectively. The pooled RR of the two prospective studies was 1.65 (95% CI, 1.30–2.08).

### 3.4. Sensitivity Analysis

**Figure 3:**
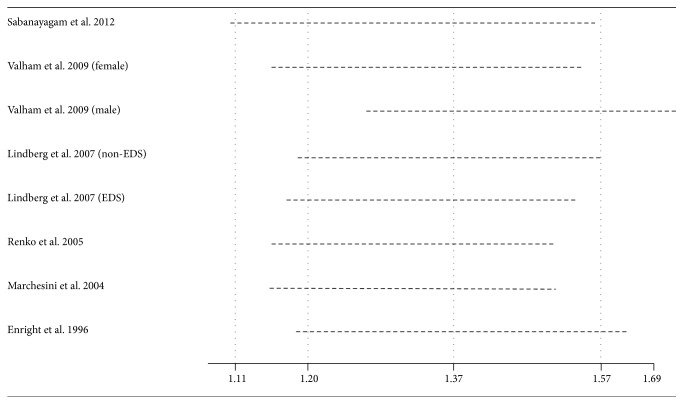
Effect of individual studies on the pooled OR for the self-reported habitual snoring and diabetes mellitus risk.

Sensitivity analyses were performed to verify the robustness of the results. These separate statistical analyses were conducted by omitting one study each time. The OR (95% CI) estimates ranged from 1.42 (1.27–1.60) to 1.54 (1.37–1.74). The data suggested that no individual study affected the results in the meta-analysis (Figure 3).

### 3.5. Publication Bias

**Figure 4:**
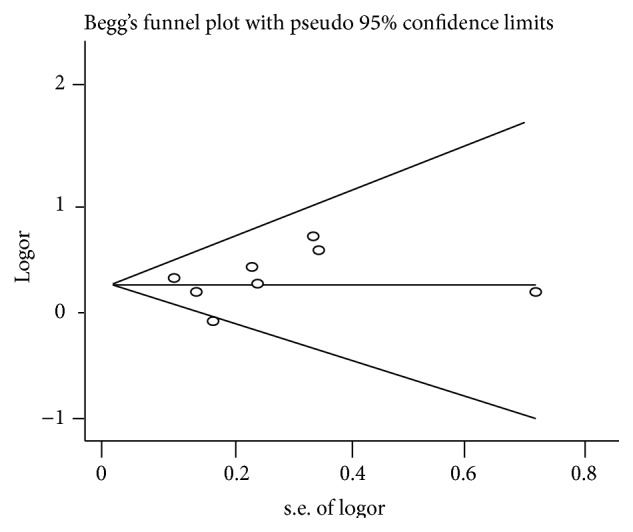
Funnel plot of the association between the self-reported habitual snoring and diabetes mellitus risk.

To assess publication bias among the included cross-sectional studies, Begg's rank correlation tests and Egger linear regression tests were performed; the *p* value of these two tests was 0.386 and 0.648, respectively. No asymmetrical funnel plots were found, as shown in Figure 4.

## 4. Discussion

This meta-analysis of six cross-sectional and two prospective cohort studies with a total of 101,246 participants revealed a statistically significant association between self-reported habitual snoring and DM. When stratified according to gender, there was a strong association between snoring and DM in females, but not in males.

Snoring affects both genders, including 23% of middle-aged males and 10% of females [24]. Obese subjects have a higher proportion of snoring than do the nonobese general population (45% versus 35%) [25]. Interestingly, the current meta-analysis stratified the studies according to gender, which revealed that females with habitual snoring were found to be associated with DM (OR, 1.59; 95% CI, 1.20–2.11). Conversely, there was no statistical significance in males (OR, 0.89; 95% CI, 0.65–1.22). Two prospective cohort studies also revealed the same phenomenon: RR 1.63 (1.29–2.07) in females and OR 1.06 (0.36–3.1) in nonobese males [1, 23]. The reasons why females who snore habitually are more associated with DM than males are as follows: (1) the average age of women who suffer sleep disordered breathing was older than males; (2) polycystic ovary syndrome (PCOS) is a common condition in premenopausal women (7%); women who have PCOS are more likely to develop into DM and sleep apnea [18]. Other variables such as age and BMI could not be included in further subgroup analysis due to limited data in the included studies.

Currently, many snorers ignore the adverse health effects that are caused by snoring. Although the relationship between habitual snoring and DM has been established, the exact mechanisms for this association have not been elucidated completely. Snoring is a sign of compromised upper airways and OSA and is characterized by the recurrent collapse of respiratory structures during breathing while asleep [26]. Nocturnal intermittent hypoxia and hypercapnia might contribute to increased sympathetic nervous activity and increased oxidative stress, which finally lead to insulin resistance [27]. The activation of proinflammatory cytokine production also plays an important role in the progression of snoring-induced DM [28]. Snoring was also closely associated with atherosclerosis and cardiovascular diseases [29], which might be prone to DM development. Due to these adverse effects, self-reported snoring might be useful as a low-cost and noninvasive indicator during the screening of persons who are prone to DM, particularly in developing countries.

Several limitations must be addressed when interpreting the results of the current meta-analysis. First, the evidence in our meta-analysis is based mostly on observational studies; therefore, no inherent causality was addressed. Second, the definition of habitual snoring and the diagnostic criteria used for DM were inconsistent among the included studies; therefore, the misclassification of snoring and DM is inevitable. Third, unmeasured confounding variables could have affected the association between snoring and DM. Although we used the most fully adjusted estimates in each study, we cannot exclude the possibility that other confounders might have affected the association. Fourth, snoring might be an early marker of unmeasured OSA. However, none of the included studies use standard method to exclude OSA. That is to say, whether snoring without OSA might be prone to DM was undetermined. Finally, most of the included studies were cross-sectional, and so additional prospective cohort studies should be performed to explore the etiology of snoring-induced DM. Despite these limitations, this is the first meta-analysis to address the association between self-reported habitual snoring and DM, there was no heterogeneity, and sensitivity analysis revealed that the results were robust.

In conclusion, the current meta-analysis pooled eight studies and revealed that habitual snoring was associated with DM. However, it remains unclear how much of the susceptibility could be attributed to OSA. Additional well-designed studies with a larger sample size are warranted to validate these findings.

## Figures and Tables

**(a):** 

First author Year	Country	Source and study type	Sample size	Female (%)	Age range	OR or RR (95% CI)	Adjusted variables
Sabanayagam 2012 [17]	America	Population-based Cross-sectional	6522	48.8	20–85	1.44 (1.16–1.79)^a^	Age, sex, ethnicity, education, smoking, alcohol, physical activity, BMI, depression, SBP, CRP, and TC
Valham 2009 [18]	Sweden	Population-based Cross-sectional	7905	51.2	25–79	Female: 1.58 (1.02–2.44)^a^ Male: 0.92 (0.64–1.33)^a^	Smoking, age, BMI, and waist circumference
Renko 2005 [19]	Finland	Population-based Cross-sectional	593	58.7	61–63	1.93 (1.04–3.57)^a^	Age, weight gain, smoking, alcohol dependence, and physical inactivity
Marchesini 2004 [20]	Italy	Population-based Cross-sectional	1890	78	20–65	1.31 (0.95–1.81)^a^ Female: 1.62 (1.11–2.36)^a^ Male: 0.81 (0.45–1.50)^a^	Age, sex, and BMI
Lindberg 2007 [21]	Sweden	Population-based Cross-sectional	6779	100%	20–99	No EDS: 1.36 (0.87–2.13)^a^ EDS: 1.82 (0.97–3.43)^a^	Age, BMI, smoking, physical activity, and alcohol dependency
Enright 1996 [22]	America	Population-based Cross-sectional	5201	57%	≥65	Women: 1.34 (0.10–1.65)^a^	Age and being married
Al-Delaimy 2002 [23]	America	Population-based Prospective cohort	69852	100%	40–65	1.63 (1.29–2.07)^b^	Age, high TC, high BP, smoking, BMI, physical activity, alcohol use, postmenopausal hormone use, family history of diabetes, sleeping position, sleep time, years of shift-work, and WHR
Elmasry 2000 [1]	Sweden	Population-based Prospective cohort	2504	0%	30–69	Nonobese: 1.06 (0.36–3.1)^a^ Obese: 7.0 (2.9–16.9)^a^	Age, weight gain, smoking, alcohol dependence, and physical inactivity

**(b):** 

Definition of snoring	Assessment/definition of DM	Presence of comorbidities	NOS score
Questionnaire answer: never or rare, occasionally as nonhabitual snorers; frequently as habitual snorers	Serum glucose ≥ 126 mg/dL after fasting for a minimum of 8 hours, a plasma glucose ≥ 200 mg/dL for those who fasted <8 hours or HbA1c ≥ 6.5%, a self-reported DM or current use of oral hypoglycemic medication or insulin	(—)	7
Questionnaire answer: always or often as habitual snorers; sometimes, never, or almost never as nonhabitual snorers	Questionnaire answer: ‘‘Do you suffer from DM?”	(—)	8
Questionnaire answer: those who reported snoring every or almost every night were classified as habitual snorers	Previously diagnosed DM, OGTT according to WHO criteria in 1998	(—)	7
Questionnaire answer: occasional or habitual as habitual snorers	Previously diagnosed DM, OGTT according to WHO criteria in 1998	Hypertension, hyperlipidemia	7
Questionnaire answer: how often they snored using a five-point scale; snoring was defined as a score of 3–5	Questionnaire answer: ‘‘Do you have diabetes?” and/or attended regular medical examinations for diabetes	Hypertension	7
Questionnaire answer: yes or no or don't know; yes as habitual snorers	History of DM, current use of insulin or oral hypoglycemic medication, fasting glucose ≥ 140 mg/dL, or 2-hour postload glucose ≥ 200 mg/dL.	Hypertension, carotid disease, and arthritis	7
Questionnaire answer: regularly as habitual snorers; occasionally or never	Classic symptoms associated with an elevated plasma glucose level or no symptoms, but at least two elevated plasma glucose values on different occasions; or treatment with hypoglycemic medication	(—)	8
Questionnaire answer: a five-point scale: ≥4 was defined as habitual snorers; ≦3 was defined as nonhabitual snorers	Questionnaire: self-report DM or confirmed by medical records	(—)	8

OR, odds ratio; RR, risk ratio; BMI, body mass index; SBP, systolic blood pressure; CRP, C reactive protein; TC, cholesterol; BP, blood pressure; DM, diabetes mellitus; HbA1c, glycosylated hemoglobin; OGTT, oral glucose tolerance test; WHO, World Health Organization; NOS: Newcastle-Ottawa scale. Note: a means studies report OR, while b means study reports RR.
